# Quantitative characterizations of the cholesterol-related pathways in the retina and brain of hamsters

**DOI:** 10.1016/j.jlr.2023.100401

**Published:** 2023-06-15

**Authors:** Natalia Mast, Nicole El-Darzi, Yong Li, Irina A. Pikuleva

**Affiliations:** Department of Ophthalmology and Visual Sciences, Case Western Reserve University, Cleveland, OH, USA

**Keywords:** hamster, retina, brain, cholesterol biosynthesis, 24-hydroxycholesterol, deuterium enrichment, deuterated water, deuterated cholesterol

## Abstract

The retina and brain are separated from the systemic circulation by the anatomical barriers, which are permeable (the outer blood-retinal barrier) and impermeable (the blood-brain and inner blood-retina barriers) to cholesterol. Herein we investigated whether whole-body cholesterol maintenance affects cholesterol homeostasis in the retina and brain. We used hamsters, whose whole-body cholesterol handling is more similar to those in humans than in mice, and conducted separate administrations of deuterated water and deuterated cholesterol. We assessed the quantitative significance of the retinal and brain pathways of cholesterol input and compared the results with those from our previous studies in mice. The utility of the measurements in the plasma of deuterated 24-hydroxycholesterol, the major cholesterol elimination product from the brain, was investigated as well. We established that despite a sevenfold higher serum LDL to HDL ratio and other cholesterol-related differences, in situ biosynthesis remained the major source of cholesterol for hamster retina, although its quantitative significance was reduced to 53% as compared to 72%–78% in the mouse retina. In the brain, the principal pathway of cholesterol input was also the same, in situ biosynthesis, accounting for 94% of the total brain cholesterol input (96% in mice); the interspecies differences pertained to the absolute rates of the total cholesterol input and turnover. We documented the correlations between deuterium enrichments of the brain 24-hydroxycholesterol, brain cholesterol, and plasma 24-hydroxycholesterol, which suggested that deuterium enrichment of plasma 24-hydroxycholesteol could be an in vivo marker of cholesterol elimination and turnover in the brain.

The retina and brain are constituents of the central nervous system (CNS) with the retina being a sensory tissue in the back of the eye. The retina captures light photons and converts them into electrical signals for subsequent transmission to the brain. Anatomically and developmentally, the retina is an extension of the CNS and is often called a window to the brain (1). The retina and brain are mostly composed of neurons and glial cells and are separated from the systemic circulation by the barriers, the blood–brain barrier (BBB) and blood–retinal barriers (BRB, inner and outer that face the intraretinal and choroidal vascular networks, respectively) (2). Cholesterol cannot cross the BBB. Hence, most of the brain cholesterol is synthesized in situ and is eliminated enzymatically by cytochrome P450 46A1 (CYP46A1). CYP46A1 converts cholesterol to 24-hydroxycholesterol (24HC) (3, 4, 5), which is then fluxed into the systemic circulation for subsequent metabolism in the liver (4). In the retina, only the inner BRB is impermeable to cholesterol, and cholesterol enters the retina through the outer BRB via receptor-mediated uptake of different types of cholesterol-containing lipoprotein particles in the systemic circulation (6). In addition, the retina can synthesize cholesterol locally (7, 8, 9). Cholesterol removal from the retina includes metabolism to 24HC by CYP46A1, C27-oxygenated sterols (27-hydroxycholesterol [27HC], cholestenoic acid [27COOH], or 7α-hydroxy-3-oxo-4-cholestenoic acid [7HCA]) by CYP27A1 as well as integration into lipoprotein particles (10, 11, 12, 13, 14). Both the brain and the retina contain cholesterol mainly in unesterified form (6).

Because of the BBB, cholesterol homeostasis in the brain is thought to be relatively independent of that in the whole body (3). Yet it is not clear how exposure to cholesterol in the systemic circulation affects cholesterol homeostasis in the retina. Hence, studies are required to quantitatively characterize cholesterol input to the retina in animal species with significant differences in their whole-body cholesterol maintenance and blood lipid profile. Besides mice, the most common laboratory animals could be hamsters, whose overall cholesterol handling is more similar to that in humans than in mice (15).

Indeed, in hamsters and humans, the rate of whole-body cholesterol biosynthesis (40 and 10 mg/day/kg, respectively, Table 1) is not as high as in mice (160 mg/day/kg, Table 1) (16). Hence the absolute value of the liver contribution to whole-body cholesterol biosynthesis is also not as high as in mice, despite the relative values being similar (40%, 35%, and 10% in mice, hamsters, and humans respectively, Table 1). Second, in contrast to mice, dietary cholesterol does not upregulate the bile acid biosynthesis (i.e., enzymatic cholesterol degradation in the liver) in hamsters and humans (17, 18). Therefore, unlike mice, hamsters and humans are not resistant to dietary-induced atherosclerosis (19, 20). Third, cholesterol ester transfer protein is expressed in hamsters and humans but not mice (21, 22). As a result, hamsters and humans carry much higher amounts of cholesterol in the systemic circulation on low-density lipoprotein particles (LDL) than high-density lipoprotein particles (HDL) (Table 1), which supply and remove, respectively, cholesterol excess from different organs (30). Finally, hamsters and humans differ from mice in how they edit the gene for apolipoprotein B (*Apob*), which is expressed in the intestine and liver. Hamsters and humans have only intestinal *Apob* editing and thus express both short (APOB48) and long (APOB100) apolipoprotein isoforms, with the former lacking the LDL receptor-binding domain (23, 24). Mice edit *Apob* in both the liver and intestine and therefore mainly produce the APOB48 isoform. Particles that contain APOB100 (LDL) are taken up by cells via the LDL receptor-mediated endocytosis, whereas particles, which contain APOB48 (chylomicrons), are processed via a different mechanism, which clears them from the systemic circulation faster than the APOB100-containing LDL (31, 32).

**Table 1:** A comparison of the whole-body cholesterol maintenance in mice, hamsters, and humans

Parameter	Mice	Hamsters	Humans	Ref.
Whole body cholesterol biosynthesis, mg/day/kg	160	40	10	(16)
Liver contribution to whole body cholesterol biosynthesis, %	40	35	10	(16)
Upregulation of bile acid biosynthesis by dietary cholesterol	Yes	No	No	(17, 18)
Resistance to dietary-induced atherosclerosis	Yes	No	No	(19, 20)
Expression of CETP	No	Yes	Yes	(21, 22)
*Apob* editing, main apolipoprotein isoform	Hepatic & intestinal, APOB48	Intestinal, APOB100 & APOB48	Intestinal, APOB100 & APOB48	(23, 24)
Serum LDL to HDL ratio	1 : 14	∼1 : 2	2.5-3.0 : 1^a^	(21, 25, 26, 27, 28, 29)
The ratio of the retinal in situ cholesterol biosynthesis to tissue uptake from the systemic circulation, %	72-78 to 22-28	Unknown^b^	Unknown	(9)
The ratio of the brain in situ cholesterol biosynthesis to tissue uptake from the systemic circulation, %	96 to 4	Unknown^b^	Unknown	(9)

a In normolipidemic subjects who do not take a cholesterol-lowering medication.

b Until the present work.

Previously, we developed a methodology to quantify the relative and absolute contributions of in situ biosynthesis and cholesterol uptake from the systemic circulation to the total brain and retinal cholesterol input (9). This was done by separately putting mice on deuterated drinking water (D_2_O) and deuterated dietary cholesterol (D_7_-cholesterol) and then calculating total tissue cholesterol input, tissue uptake of cholesterol from the systemic circulation, and the difference between the two, representing in situ biosynthesis. We found that in mice, in situ biosynthesis was the major source of cholesterol for both organs accounting for 97% of the cholesterol input to the brain and 72%–78% of the cholesterol input to the retina (9, 33). Herein, we applied our methodology to hamsters to quantitatively characterize their retinal and brain cholesterol input. In addition, we investigated cholesterol output from hamster brains. Collectively, the data obtained provided novel insights into interspecies differences in retinal and brain cholesterol maintenance and suggested a non-invasive in vivo approach for studies of cholesterol elimination and turnover in the brain.

## Materials and Methods

### Reagents

[25,26,26,26,27,27,27-^2^H_7_]Cholesterol (D_7_-cholesterol) was purchased from Cambridge Isotope Laboratories Inc., [25,26,26,26,27,27,27-^2^H_7_]-sitosterol (D_7_-sitosterol) was from Toronto Research Chemicals Inc. [1,2,5,6- ^2^H_4_]Lathosterol (D_4_-lathosterol), [26,26,26,27,27,27-^2^H_6_]desmosterol (D_6_-desmosterol), [25,26,26,26,27,27,27-^2^H_7_]24HC and [26,26,26,27,27-^2^H_5_]27HC were from CDN Isotopes (Pointe-Claire, Canada), 23,24-bisnor-5-cholenic acid-3β-ol from Steraloids (Newport, RI). All other chemicals, including unlabeled cholesterol and D_2_O, were purchased from MilliporeSigma. Regular rodent chow (5P75-5P76 Prolab Isopro RMH 3000), which we called normal diet (ND) was from LabDiet and contained 0.02% cholesterol (w/w) and 5.4% fat (w/w). Solutions for hamster oral gavage were prepared by dissolving cholesterol powder (1.0 g) in peanut oil (32 ml, Planters)) to reach the final 3.3% cholesterol concentration. Either unlabeled or D_7_-cholesterol were used, hence the fat- and cholesterol-containing oral gavages, which utilized these solutions were called (FCCOG or D_7_-FCCOG, respectively).

### Animals

**Fig. 1:**
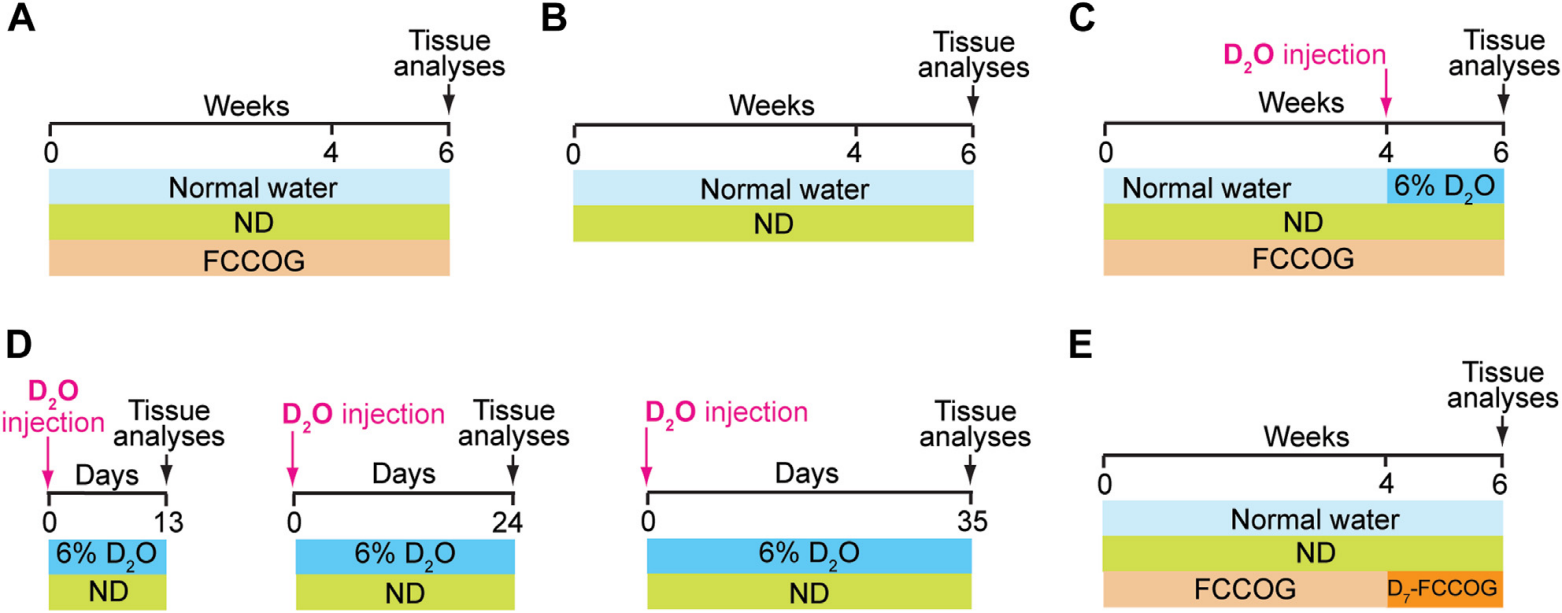
Experimental paradigms for different measurements. A: Tissue sterol levels in hamsters on a normal diet (ND) plus fat- and cholesterol-containing oral gavage (FCCOG). B: Tissue sterol levels in mice on ND. C: Tissue cholesterol input comprised of in situ biosynthesis and uptake of the synthesized cholesterol from the systemic circulation. D: Relative deuterium enrichment of 24-hydroxycholesterol. E: Tissue uptake of dietary cholesterol. D_2_O, deuterated water; D_7_-FCCOG, FCCOG containing deuterated cholesterol.

Only male Golden Syrian hamsters (Charles River, strain code: 049) were used as our prior study did not reveal any sex-based differences in the hamster serum lipid profile and retinal content of different sterols (25). Animals were purchased at 8 weeks of age and were housed one animal per cage until the age of 6 months when they became mature and their retinal cholesterol homeostasis reached a steady state (25). During the growth period and until used in the experiment, animals were maintained on a standard 12-h light (approximately 10 lux) dark cycle and were fed standard rodent chow with normal water being provided ad libitum. The subsequent assignment of 6-month-old hamsters to experimental groups was random and utilized the pool of all available animals; the sample size was based on previous experience. A total of seven independent hamster studies were performed with the experimental design of each being schematically described in the Fig. 1 panels. At the end of each experiment, animals were fasted overnight for 12 h as described (25). Briefly, their cheek pouches were emptied with rubber-tipped forceps, and their food and bedding were removed. The next morning hamsters were deeply sedated with a 200 mg/kg ketamine and 10 mg/kg xylazine bolus injection (Patterson Veterinary, Greeley, CO, USA, 07-890-8598 and 07-808-1947, respectively). Blood was withdrawn via cardiac puncture, and animals were perfused through the heart with 50 ml of phosphate-buffered saline, 1.5 ml/min. Hamsters were then decapitated by guillotine, and their eyes and brain were excised. The eyes were dissected to obtain the retina, which contained the neural retina and retinal pigment epithelium, and the olfactory bulbs were removed from the brains for the latter to only contain cerebrum, cerebellum, and lower brain stem. The brains were next dissected along the midline to obtain two hemispheres, which, along with the retinas, were individually flash-frozen in liquid nitrogen and stored at −80^o^C until subsequent processing. The investigators were not blinded with respect to treatment as they were involved in both animal treatment and subsequent tissue isolation. All tissue assessments were quantitative and hence could not be affected by investigators’ bias as all data were used and apparent outliers were not excluded. All animal handling and experiments were approved by Case Western Reserve University IACUC and conformed to the recommendations of the American Veterinary Association Panel on Euthanasia.

### Administration of oral gavage

Oral gavage was used to deliver increased amounts of fat and cholesterol as compared to ND. Hamsters were put for 6 weeks on normal water and ND and received FCCOG every day from noon to 1 pm (Fig. 1A). The volume of oral gavage varied from 0.9 ml to 1.1 ml, depending on animal weight (from 168 g to 189 g), and represented a daily intake (including ND) of ∼0.15% (w/w) cholesterol and ∼9.2% (w/w) fat, assuming that an average hamster eats 10–12 g of chow per day per 100 g of body weight (34). This oral gavage group was compared to a group of same-age hamsters, which received normal water and ND only (Fig. 1B).

### Administration of D_2_O to measure total tissue cholesterol input

Hamsters were put for 6 weeks on ND and daily FCCOG and received normal water during the first 4 weeks of the FCCOG administration (Fig. 1C). Then, hamsters were injected intraperitoneally with 3.5–4.0 ml D_2_O (equal to ∼3.5% of hamster body water), and normal water was replaced with 6% D_2_O (v/v) for the next 2 weeks until animals were euthanized.

### Administration of D_2_O to characterize plasma deuterated 24HC as a marker of brain cholesterol turnover

Three groups of hamsters were used, which were all on ND and received normal water prior to a single intraperitoneal D_2_O injection (3.5–4.0 ml) (Fig. 1D). Then, normal water was replaced with 6% D_2_O (v/v), which was administered for 13 days to the first group, 24 days to the second group, and 35 days to the third group until animals were euthanized.

### Administration of D_7_-cholesterol to measure tissue uptake of dietary cholesterol

Hamsters were on normal water and ND and received daily FCCOG for the first 4 weeks of treatment and D_7_-FCCOG for the next 2 weeks of treatment until animals were euthanized (Fig. 1E).

### Tissue isolation and processing

After euthanasia and blood withdrawal, serum was prepared by keeping the blood samples at room temperature for 30 min and pelleting the clot by centrifugation at 1,500 *g* for 10 min. Plasma was prepared by keeping the blood samples at room temperature for 30 min in Eppendorf tubes coated with 0.5 M aqueous EDTA. Samples were then centrifuged at 2,000 *g* for 10 min, and the supernatant obtained represented plasma. Frozen brain hemispheres and retinas were homogenized and processed as described for mice (9, 10, 35). Left hemispheres were always used for the quantification of unlabeled sterols, and right hemispheres were always used for the determination of deuterium sterol enrichments or D_7_-cholesterol incorporation. Deuterated sterol analogs were added to tissue homogenates for cholesterol, lathosterol, desmosterol, 24HC, and 27HC quantifications; 23,24-bisnor-5-cholenic acid-3β-ol was used for 27COOH and 7-HCA quantifications. For samples from hamsters that received D_7_-FCCOG, D_7_-sitosterol was added as an internal standard for cholesterol quantifications. Serum was sent to the IDEXX Laboratories for measurements of total, HDL, and LDL cholesterol, and triglycerides. Sterol quantifications were conducted in-house by gas chromatography-mass spectrometry as described (9, 10, 33).

### Total tissue cholesterol input

This was measured after D_2_O administration (Fig. 1C) as previously described for mice (9). The fragment ions of cholesterol at *m/z* 368→372 were monitored followed by the correction for the natural abundance of cholesterol mass isotopomers (36) and the calculation of the average number of deuterium atoms incorporated per cholesterol molecule. Then, the abundance of each ion from m/z 368 to m/z 372 was divided by the sum of the abundance of all sterol ions and multiplied by 100. The value for m/z 368 was next multiplied by 0, for m/z 369 was multiplied by 1, for m/z 370 was multiplied by 2, for m/z 371 was multiplied by 3, and for m/z 372 was multiplied by 4 and summed up. This summary value was then divided by 22, the maximal deuterium incorporation into cholesterol molecule (37), and by animal deuterium body water enrichment. The resulting value represented the percentage of deuterium cholesterol enrichment in each tissue and reflected a sum of tissue cholesterol biosynthesis and cholesterol uptake from the systemic circulation over 2 weeks.

### Tissue uptake of dietary cholesterol

The tissue appearance of D_7_-cholesterol in samples from hamsters on D_7_-FCCOG (Fig. 1E) was measured based on the fragment ion at *m/z* 375 and was presented as the percentage from the sum of tissue unlabeled (*m/z* 368) and D_7_-cholesterol (*m/z* 375) (9).

### Deuterium whole-body water enrichment

This was measured as previously described for mice (9) after the isotopic exchange with acetone of the serum of hamsters, which received D_2_O.

### Rates of tissue cholesterol uptake and local biosynthesis

These rates were calculated as previously described for mice (9, 33) using the following equations.

(1) $$\begin{array}{c} {\text{Tissues}\;\text{relative}\;\text{D}}_{7}-\text{cholesterol}\\ \text{u}\text{p}\text{t}\text{a}\text{k}\text{e}\;\left(2\;\text{weeks},\%\right) \end{array}\;=\;\frac{{\text{Tissue}\;\text{relative}\;\text{D}}_{7}-\text{cholesterol}\;\left(\%\right)}{{\text{Serum}\;\text{relative}\;\text{D}}_{7}-\text{cholesterol}\;\left(\%\right)}\;\times \;100$$

(2) $$\begin{array}{c} \text{Tissue}\;\text{relative}\;\text{deuterium}\\ \text{cholesterol}\;\text{enrichment}\\ \text{from}\;\text{uptake}\;\left(2\;\text{weeks},\%\right) \end{array}=(\begin{array}{c} \begin{array}{c} \text{T}\text{i}\text{s}\text{s}\text{u}\text{e}\;\text{r}\text{e}\text{l}\text{a}\text{t}\text{i}\text{v}\text{e}\\ {\text{D}}_{7}-\text{cholesterol} \end{array}\\ \text{u}\text{p}\text{t}\text{a}\text{k}\text{e}\;\left(2\;\text{w}\text{e}\text{e}\text{k}\text{s},\%\right) \end{array}\times \begin{array}{c} \text{S}\text{e}\text{r}\text{u}\text{m}\;\text{r}\text{e}\text{l}\text{a}\text{t}\text{i}\text{v}\text{e}\\ \text{d}\text{e}\text{u}\text{t}\text{e}\text{r}\text{i}\text{u}\text{m}\;\text{c}\text{h}\text{o}\text{l}\text{e}\text{s}\text{t}\text{e}\text{r}\text{o}\text{l}\\ \text{e}\text{n}\text{r}\text{i}\text{c}\text{h}\text{m}\text{e}\text{n}\text{t}\;\left(2\;\text{w}\text{e}\text{e}\text{k}\text{s},\%\right) \end{array})/\;100$$

(3) $$\begin{array}{c} \begin{array}{c} \text{Tissue}\;\text{relative}\\ \text{cholesterol}\\ \text{biosynthesis} \end{array}\\ \left(2\;\text{weeks},\%\right) \end{array}=\begin{array}{c} \begin{array}{c} \text{Tissue}\;\text{relative}\\ \text{deuterium}\;\text{cholesterol}\\ \text{enrichment} \end{array}\\ \left(2\;\text{weeks},\%\right) \end{array}\;–\;\left(\begin{array}{c} \begin{array}{c} \text{Tissue}\;\text{relative}\\ {\text{D}}_{7}-\text{cholesterol} \end{array}\\ \text{u}\text{ptake}\\ \left(2\;\text{weeks},\%\right) \end{array}\times \begin{array}{c} \begin{array}{c} \begin{array}{c} \text{Serum}\;\text{relative}\\ \text{deuterium}\\ \text{cholesterol} \end{array}\\ \text{enrichment} \end{array}\\ \left(2\;\text{weeks},\%\right) \end{array}\right)\;/\;100$$

(4) $$\begin{array}{c} \begin{array}{c} \text{Absolute}\;\text{rate}\\ \text{of}\;\text{cholesterol}\;\text{input} \end{array}\\ \left(\text{mg}/\text{day}/\text{g}\;\text{wet}\;\text{tissue}\right) \end{array}=\begin{array}{c} \begin{array}{c} \text{Cholesterol}\\ \text{c}\text{o}\text{n}\text{c}\text{e}\text{n}\text{t}\text{r}\text{a}\text{t}\text{i}\text{o}\text{n} \end{array}\\ \left(\text{m}\text{g}/\text{g}\;\text{w}\text{e}\text{t}\;\text{t}\text{i}\text{s}\text{s}\text{u}\text{e}\right) \end{array}\times \left(\begin{array}{c} \begin{array}{c} \text{R}\text{e}\text{l}\text{a}\text{t}\text{i}\text{v}\text{e}\\ {\text{D}}_{7}-\text{cholesterol} \end{array}\\ \text{u}\text{p}\text{t}\text{a}\text{k}\text{e}\\ \left(\text{w}\text{e}\text{e}\text{k},\%\right) \end{array}+\begin{array}{c} \begin{array}{c} \begin{array}{c} \text{R}\text{e}\text{l}\text{a}\text{t}\text{i}\text{v}\text{e}\\ \text{c}\text{h}\text{o}\text{l}\text{e}\text{s}\text{t}\text{e}\text{r}\text{o}\text{l} \end{array}\\ \text{b}\text{i}\text{o}\text{s}\text{y}\text{n}\text{t}\text{h}\text{e}\text{s}\text{i}\text{s} \end{array}\\ \left(\text{w}\text{e}\text{e}\text{k},\%\right) \end{array}\right)/100/7$$

(5) $$\begin{array}{c} \text{In}\;\text{situ}\;\text{b}\text{i}\text{o}\text{s}\text{y}\text{n}\text{t}\text{h}\text{e}\text{s}\text{i}\text{s}\\ \left(\text{mg}/\text{day}/\text{g}\;\text{wet}\;\text{tissue}\right) \end{array}=\begin{array}{c} \text{Cholesterol}\;\text{concentration}\\ \left(\text{mg}/\text{g}\;\text{wet}\;\text{tissue}\right) \end{array}\times \begin{array}{c} \text{Relative}\;\text{cholesterol}\\ \text{biosynthesis}\;\left(\text{weeks},\%\right) \end{array}/100/7$$

(6) $$\begin{array}{c} \text{Uptake}\;\text{from}\;\text{blood}\\ \left(\text{mg}/\text{day}/\text{g}\;\text{wet}\;\text{tissue}\right) \end{array}=\begin{array}{c} \text{Cholesterol}\;\text{concentration}\\ \left(\text{mg}/\text{g}\;\text{wet}\;\text{tissue}\right) \end{array}\times \begin{array}{c} {\text{Relative}\;\text{D}}_{7}-\text{cholesterol}\\ \text{u}\text{p}\text{t}\text{a}\text{k}\text{e}\;\left(\text{week},\%\right) \end{array}\;/100/7$$

(7) $$\begin{array}{c} \text{Cholesterol}\;\text{turnover}\\ \left(\text{days}\right) \end{array}=\begin{array}{c} \text{Cholesterol}\;\text{concentration}\\ \left(\text{mg}/\text{g}\;\text{wet}\;\text{tissue}\right) \end{array}/\;\begin{array}{c} \text{Absolute}\;\text{rate}\;\text{of}\;\text{cholesterol}\\ \text{input}\;\left(\text{mg}/\text{day}/\text{g}\;\text{wet}\;\text{tissue}\right) \end{array}$$

### Deuterated 24HC as a marker of brain cholesterol turnover

Deuterium incorporation into cholesterol (m/z 368→372) and 24HC (m/z 503→506) was assessed after D_2_O administration for 13, 24, and 35 days (Fig. 1D) and data correction for the natural abundance of the sterol mass isotopomers. Then, the abundance of each ion (from m/z 368 to m/z 372 for cholesterol and from m/z 503 to m/z 506 for 24HC) was divided by the sum of the abundance of all sterol ions and multiplied by 100. The values for m/z 368 or m/z 503 were next multiplied by 0, for m/z 369 and m/z 504 were multiplied by 1, for m/z 370 and m/z 505 were multiplied by 2, for m/z 371 and m/z 506 were multiplied by 3, and for m/z 372 was multiplied by 4 and summed up. These relative values (%) for total deuterium [^2^H] incorporation into cholesterol and 24HC were then divided by 22 and animal’s deuterium body water enrichment and represented relative deuterium sterol enrichments (%).

### Statistics

All data represent the mean ± S.D. of the measurements in individual hamsters; the number of animals (n) is indicated in each figure. Data were analyzed by a two-tailed, unpaired Student’s *t* test using GraphPad Prism software (GraphPad Software). Statistical significance was defined as ∗*P* ≤ 0.05, ∗∗*P* ≤ 0.01, and ∗∗∗*P* ≤ 0.001.

## Results

### Effect of increased cholesterol and fat intake on serum lipid profile and tissue sterol concentrations

**Fig. 2:**
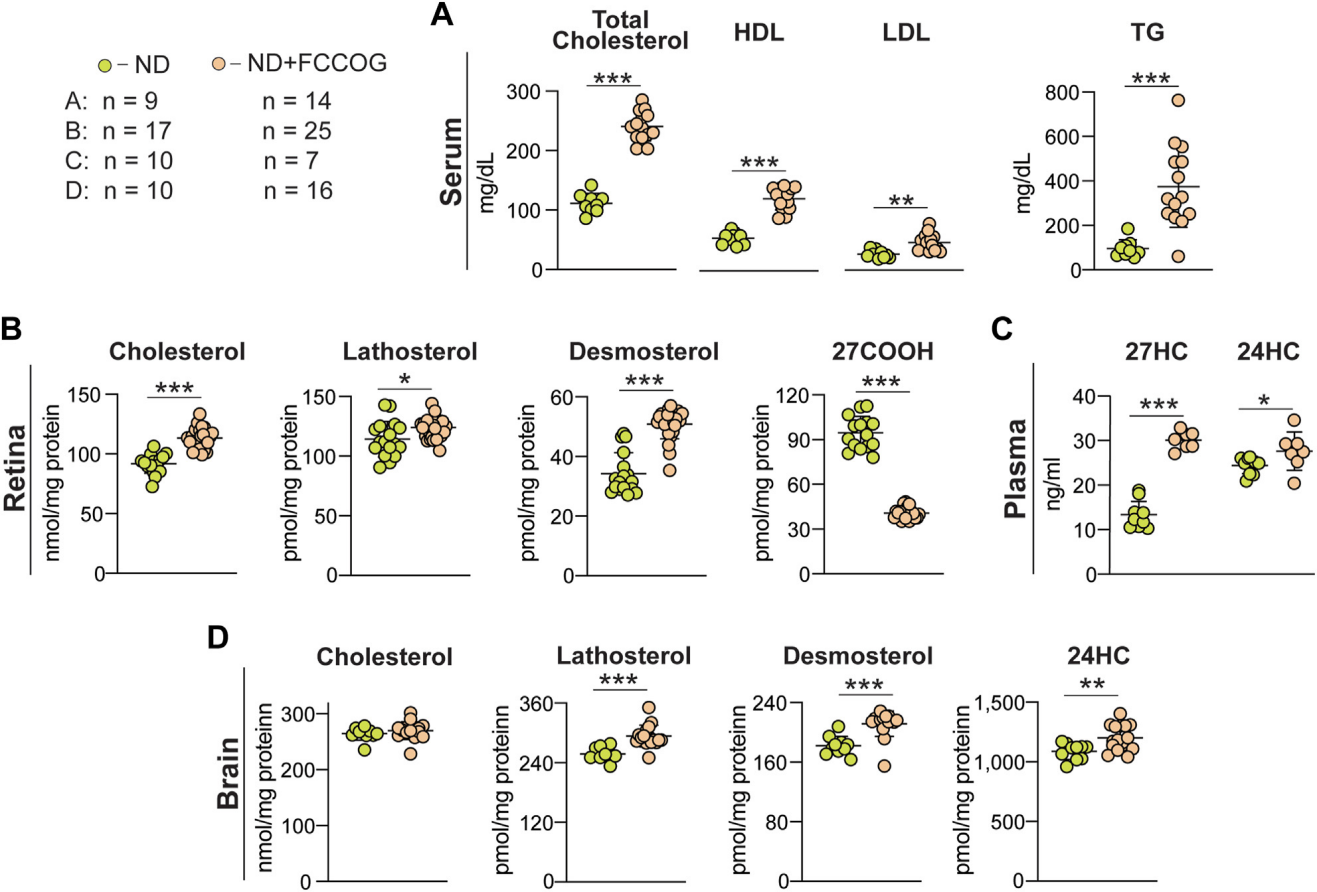
Effects of the fat- and cholesterol-containing oral gavage (FCCOG). A: Serum lipid profile measured by a clinical laboratory. B–D: Tissue sterols quantified by GS-MS. All data represent the mean ± SD of the measurements in individual hamsters; the number of animals (n) for each panel is indicated in the figure. Statistical significance was assessed by a two-tailed, unpaired Student’s *t* test. ∗*P* ≤ 0.05; ∗∗*P* ≤ 0.01; ∗∗∗*P* ≤ 0.001. 27COOH, cholestenoic acid; 27HC, 27-hydroxycholesterol; HDL, high density lipoprotein cholesterol; LDL, low density lipoprotein cholesterol; TG, triglycerides.

Our methodology uses dietary D_7_-cholesterol to quantify tissue cholesterol uptake (9). Hence, we first assessed the effect of an increased fat and cholesterol intake from 5.4% and 0.02%, respectively, provided by ND, to ∼9.2% and ∼0.15%, respectively, obtained from ND plus FCCOG (Fig. 1A, B). Administration of FCCOG led to a significant increase in the levels of all measured serum lipids (Fig. 2A). Total cholesterol was increased 2.1-fold (from 112 to 240 mg/dl), HDL cholesterol was increased 2.2-fold (from 55 to 121 mg/dl), LDL cholesterol was increased 1.7-fold (from 27 to 46 mg/dl), and the TG concentration was increased 3.9-fold (from 96 to 374 mg/dl). Nevertheless, the serum LDL to HDL cholesterol ratio was only changed from 0.49 on ND to 0.38 on ND plus FCCOG.

To assess the FCCOG effects on tissue sterol profiles, we used GC-MS and repeated the total cholesterol measurements in the serum as the initial serum lipid quantifications were conducted by a clinical laboratory (Fig. 2A). The measured total cholesterol levels in hamsters on ND and ND plus FCCOG (83 and 203 mg/dl, respectively, Table 2, line 2) were comparable with those determined by a clinical laboratory (Fig. 2A) and documented a similar more than 2-fold (2.4-fold) sterol increase.

**Table 2:** A summary of experimental data and calculations

Line #	Parameter	Serum	Retina	Brain	Used Equation
1	***ND plus FCCOG***				
2	Cholesterol concentration: serum (mg/dl), retina and brain (nmol/mg protein)	203	109	268	
3	Cholesterol concentration (mg/g wet tissue)		4.0	11.9	
4	***Treatment with Deuterated Water***				
5	Relative deuterium cholesterol enrichment (2 weeks, %)	8.6	9.9	5.8	
6	***Treatment with D***_***7-***_***Cholesterol***				
7	Relative D_7_-cholesterol enrichment (2 weeks, %)	33.5	2.75	0.12	
8	***Calculations***				
9	Tissue relative D_7_-cholesterol uptake (2 weeks, %)	ND	8.2	0.4	Equation 1
10	Tissue relative D_7_-cholesterol uptake (week, %)		4.1	0.2	
11	Tissue relative deuterium cholesterol enrichment from uptake (2 weeks, %)		0.71	0.03	Equation 2
12	Tissue relative cholesterol biosynthesis (2 weeks, %)		9.19	5.77	Equation 3
13	Tissue relative cholesterol biosynthesis (week, %)		4.60	2.88	
14	Tissue relative cholesterol input (week, %)		8.70	3.06	
15	Tissue relative cholesterol biosynthesis (week, %)		52.8	94.2	
16	Tissue relative cholesterol uptake from the serum (week, %)		47.2	5.8	
17	Absolute rate of cholesterol input (mg/day/g wet tissue)		0.050	0.052	Equation 4
18	In situ biosynthesis (mg/day/g wet tissue)		0.026	0.049	Equation 5
19	Uptake from the blood (mg/day/g wet tissue)		0.024	0.003	Equation 6
20	Cholesterol turnover (days)		80	228	Equation 7

In the retina, FCCOG led to an increase in the total cholesterol content (1.2-fold, from 92 to 113 nmol/mg protein or to 4.0 mg/g wet tissue, Table 2, line 3) as well as lathosterol content (1.1-fold, from 114 to 124 nmol/mg protein) and desmosterol content (1.5-fold, from 34 to 51 pmol/mg protein). The 27COOH content was decreased (2.3-fold, from 95 to 41 pmol/mg protein) (Fig. 2B), and the content of 24HC, 27HC, and 7HCA was below the limit of detection (1 pmol/mg protein). To interpret these data, we conducted an additional quantification of 27HC in hamster plasma to investigate whether an increase in the serum HDL concentrations as a result of FCCOG facilitated retinal 27HC efflux to the systemic circulation and hence decreased retinal 27COOH content, which is generated from 27HC (38, 39). Indeed, plasma 27HC levels were increased 2.4-fold (from 13 to 31 ng/ml) in hamsters on FCCOG (Fig. 2C), consistent with HDL being an extracellular 27HC acceptor and carrying this sterol in the systemic circulation (38, 40). These results supported the mechanism based on 27HC as a suppressor of cholesterol biosynthesis (41, 42, 43), thus explaining the increase in the levels of lathosterol and desmosterol, markers of cholesterol biosynthesis in neurons and astrocytes, respectively (33, 44), and ultimately a modest increase in the total retinal cholesterol levels (Fig. 2B).

In the brain, FCCOG did not alter total cholesterol content (268 nmol/mg protein or 11.9 mg/g wet tissue, Table 2, lines 2,3) relative to ND (264 nmol/mg protein) and modestly increased lathosterol content (1.2-fold, from 258 to 294 pmol/mg protein), desmosterol content (1.2-fold, from 182 to 211 pmol/mg protein), and 24HC content (1.1-fold, from 1088 to 1201 pmol/mg protein) (Fig. 2D). Plasma 24HC levels were increased as well (1.1-fold from 24 to 28 ng/ml, Fig. 2C) because of the brain 24HC flux into the systemic circulation (4). Thus, FCCOG elicited a coupled increase in the rates of brain cholesterol biosynthesis, metabolism, and efflux and hence the rate of brain cholesterol turnover. The 0.15% cholesterol content in ND plus FCCOG was a rigorous cholesterol challenge for hamsters, defined as 0.1%–0.3% dietary cholesterol (45). Our data suggested that this challenge led to prominent changes in the animal serum lipid profile but only modestly affected retinal and brain sterol content.

### Quantitative characterization of retinal and brain cholesterol input

**Fig. 3:**
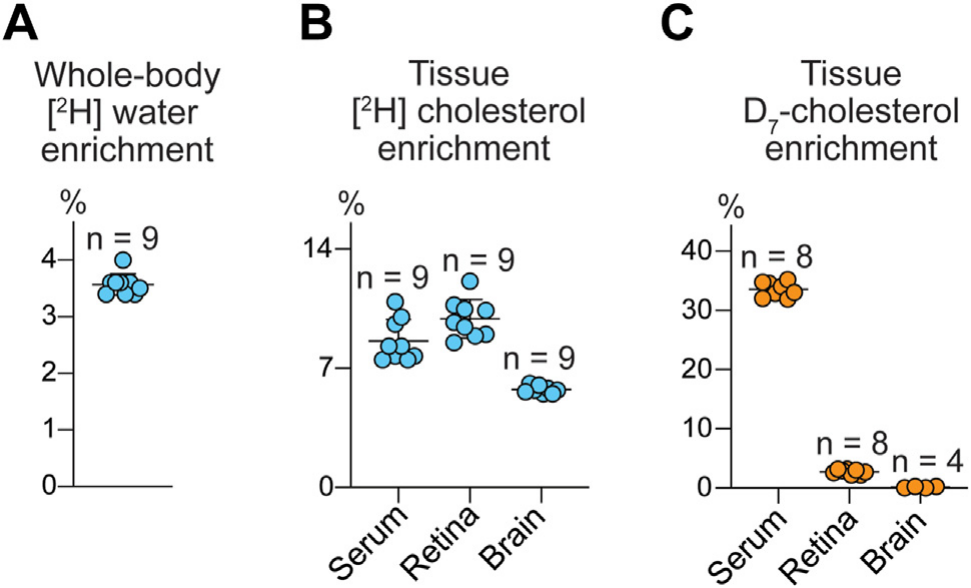
Tissue relative enrichments after 2 weeks of either D_2_O or D_7_-FCCOG administration. A: Whole-body water deuterium ([^2^H]) enrichment from D_2_O. B: Tissue [^2^H] enrichment from D_2_O. C: Tissue D_7_-cholesterol enrichment from D_7_ fat- and cholesterol-containing oral gavage FCCOG. All data represent the means ± S.D. of the measurements in individual hamsters; the number of animals (n) is indicated in each panel.

To quantify total tissue cholesterol input, we determined relative deuterium enrichment of tissue cholesterol after D_2_O administration (Fig. 1C): 8.6%, 9.9%, and 5.8% in the serum, retina, and brain, respectively (Fig. 3B and Table 2, line 5). Also, we determined relative tissue D_7_-cholesterol enrichment after D_7_-FCCOG administration (Fig. 1E) to quantify tissue uptake of cholesterol from the systemic circulation. These values were 2.75%, and 0.12% in the retina and brain, respectively; in the serum, the D_7_-cholesterol enrichment was 33.5% (Fig. 3C and Table 2, line 7).

The experimental data obtained were then used to calculate the relative tissue D_7_-cholesterol uptake (4.1% and 0.2% per week in the retina and brain, respectively, Table 2, lines 9 and 10) and relative tissue deuterium enrichment of cholesterol from uptake (0.71% and 0.03% per 2 weeks in the retina and brain, respectively, Table 2, line 11). The relative tissue cholesterol biosynthesis was also calculated (4.60% and 2.88% per week, in the retina and brain, respectively, Table 2, lines 12,13), thus enabling the calculation of the relative tissue cholesterol input (8.70% and 3.06% in the retina and brain, respectively, Table 2, line 14), which was a sum of the relative tissue D_7_-cholesterol uptake (Table 2, line 10) and relative in situ cholesterol biosynthesis (Table 2, line 13). The relative values of in situ cholesterol biosynthesis (52.8% and 94.2% in the retina and brain, respectively, Table 2, line 15) and tissue cholesterol uptake from the serum (47.2% and 5.8% in the retina and brain, respectively, Table 2, line 16) were then calculated as were the absolute tissue cholesterol input rates (0.026 and 0.024 mg/day/g wet tissue for the retinal biosynthesis and uptake and 0.049 and 0.003 mg/day/g wet tissue for the brain biosynthesis and uptake, Table 2, lines 18,19). The rates of tissue cholesterol turnover (80 and 228 days in the retina and brain, respectively, Table 2, line 20) were also determined. Thus, we obtained the dataset that was necessary for our subsequent comparative analyses of the interspecies differences in retinal and brain cholesterol maintenance (Table 3).

**Table 3:** A comparison of cholesterol input to the retina and brain in hamsters and mice

Parameter	Hamsters			Mice		
	Serum	Retina	Brain	Serum	Retina	Brain
Cholesterol concentration: serum (mg/dl); retina and brain (nmol/mg protein)	203 ± 16	109 ± 8	268 ± 16	163 ± 12	64 ± 10	271 ± 14
Cholesterol concentration (mg/g wet tissue)		4.0 ± 0.3	11.9 ± 0.8		2.5 ± 0.4	12.2 ± 0.6
Absolute rate of cholesterol input (mg/day/g wet tissue)		0.050 (100%)	0.052 (100%)		0.036 (100%)	0.117 (100%)
In situ biosynthesis (mg/day/g wet tissue)		0.026 (53%)	0.049 (94%)		0.028 (78%)	0.112 (96%)
Uptake from blood (mg/day/g wet tissue)		0.024 (47%)	0.003 (6%)		0.008 (22%)	0.005 (4%)
Tissue cholesterol turnover (days)		80	228		72	104

Data that were obtained experimentally represent the mean ± SD of the measurements in individual animals; at least 7 animals were used for each tissue quantification. Data for mice were generated previously (33).

### Deuterated 24HC as in vivo marker of brain cholesterol elimination and turnover

**Fig. 4:**
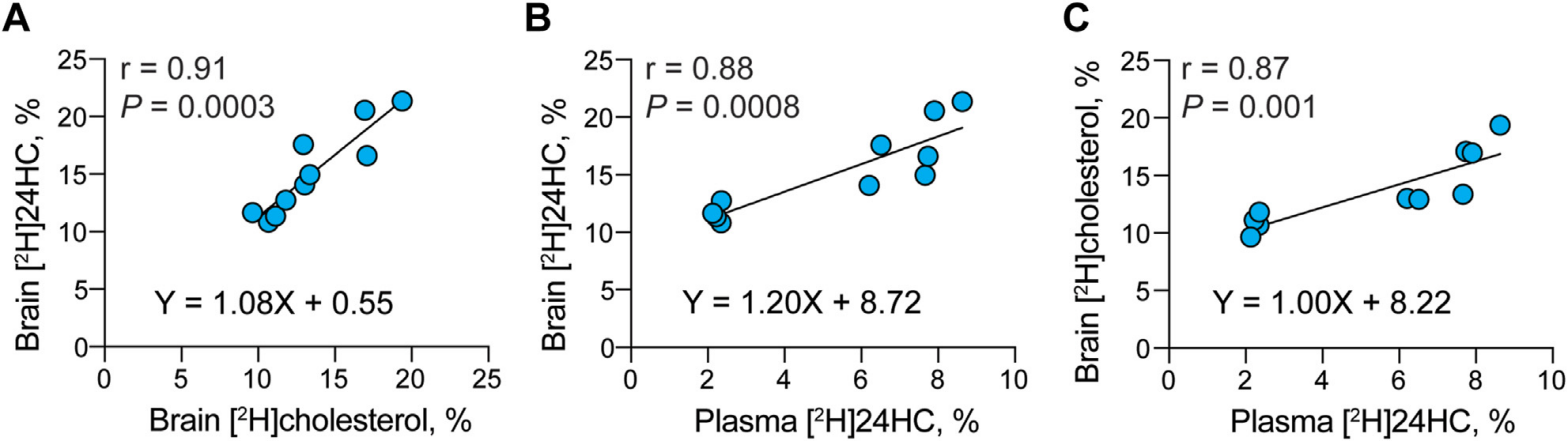
Correlation analyses. A: Relative deuterium ([^2^H]) enrichment of brain 24-hydroxycholesterol (24HC) versus relative [^2^H] enrichment of brain cholesterol. B: Relative [^2^H] enrichment of brain 24HC versus relative [^2^H] enrichment of plasma 24HC. C: Relative [^2^H] enrichment of brain cholesterol versus relative [^2^H] enrichment of plasma 24HC. Each dot represents the measurements in individual hamster, a total of 10 measurements (hamsters) in each panel: 4 animals were on D_2_O for 13 days, 3 animals were on D_2_O for 24 days, and 3 animals were on D_2_O for 35 days. A Pearson correlation coefficient and *P* value were computed to assess the linear relationship between the two variables in each panel. Data were fit by simple linear regression; the regression equations are indicated in each panel.

Plasma 24HC is used as a surrogate marker of cerebral 24HC production but its levels are affected by a number of different factors (46). Hence, we investigated the utility of deuterium 24HC enrichment in hamster plasma. Hamsters were put on deuterated water for 13, 24, and 35 days (Fig. 1D), and deuterium sterol enrichment was measured in their brain and plasma. Then, several types of analyses were carried out. In the first, deuterium enrichment of the brain cholesterol was plotted against deuterium enrichment of the brain 24HC to ascertain if there is a correlation (Fig. 4A). This correlation was indeed observed (Y=1.08X+0.5, r=0.91), thus suggesting a tight coupling between the cholesterol biosynthesis and elimination via cholesterol 24-hydroxylation and consistent with previous studies of *Cyp46a1*^*−/−*^ mice (5). In the second analysis, we tested for a correlation between deuterium enrichment of the brain 24HC and deuterium enrichment of plasma 24HC. This correlation was observed as well (Y= 1.2X + 8.7, r = 0.88, Fig. 4B), consistent with the knowledge that brain 24HC is the major source of 24HC in systemic circulation (4, 5). Finally, we analyzed for a correlation between the brain cholesterol enrichment and plasma 24HC enrichment, which was also confirmed (Y = 1.0X + 8.2, r = 0.87, Fig. 4C). Thus, despite the slow rate of the whole brain cholesterol turnover in hamsters (Table 2), it was, nevertheless, possible to detect brain production of deuterated 24HC from deuterated cholesterol and subsequent flux of deuterated 24HC to the plasma. Our measurements supported the utility of deuterated 24HC in the plasma as in vivo marker of brain cholesterol elimination and turnover.

## Discussion

The present study of hamsters and our previous characterizations of mice (9, 33) enabled for the first time quantitative comparisons of the retinal and brain cholesterol input between the two species (Table 3), having significantly different whole-body cholesterol maintenance (Table 1). Several novel insights were obtained. In the retina, the absolute rates of in situ cholesterol biosynthesis were comparable in hamsters and mice (0.026 and 0.028 mg/day/g wet tissue, respectively). Yet cholesterol uptake from the systemic circulation was different and was three times higher in the hamster retina (0.024 mg/day/g wet tissue) than mouse retina (0.008 mg/day/g wet tissue). Accordingly, total retinal cholesterol input was higher in hamsters (0.05 mg/day/g wet tissue) than in mice (0.036 mg/day/g wet tissue) as was the relative contribution of the retinal cholesterol uptake from the systemic circulation (47% in hamsters vs. 22% in mice). The retinal cholesterol content was also higher in hamsters than mice (4.0 and 2.5 mg/g wet tissue, respectively), but the retinal cholesterol turnover rates were comparable (80 days in hamsters and 72 days in mice) because of the differences in the rates of the total retinal cholesterol input. Thus, despite more than a sevenfold increase in the serum LDL to HDL ratio and other differences in the whole body cholesterol maintenance (Table 1), in situ cholesterol biosynthesis remained the major source of cholesterol for the retina in hamsters, although its quantitative significance was reduced to 53% as compared to 72%–78% in mice (9, 33).

In the brain, the absolute rates of in situ cholesterol biosynthesis were different in hamsters and mice (0.049 and 0.112 mg/day/g wet tissue, respectively) as were the absolute rates of cholesterol uptake from the systemic circulation (0.003 and 0.005 mg/day/g wet tissue, respectively). The latter, however, was very small when compared to the corresponding rates of in situ biosynthesis. Therefore, in situ cholesterol biosynthesis was the predominant source of cholesterol in both species and accounted for 94% and 96% of the total brain cholesterol input in hamsters and mice, respectively. The brain cholesterol content was similar in hamsters and mice (11.9 and 12.2 mg/g wet tissue, respectively). Yet, the brain cholesterol turnover was different and slower in hamsters (228 days) than mice (104 days) due to a slower rate of the total cholesterol input. Thus, the interspecies differences in the brain cholesterol homeostasis pertained to the absolute rates of the total cholesterol input and turnover but not the principal pathway of cholesterol input, which was the same, in situ biosynthesis.

Accumulating evidence indicates that disturbances in the retinal cholesterol homeostasis contribute to the pathogenesis of such major eye diseases as age-related macular degeneration (AMD) and diabetic retinopathy (6, 47, 48, 49, 50). Hence, our finding that the serum LDL to HDL ratio affects, at least in part, retinal cholesterol uptake from the systemic circulation raises a question of whether this uptake is even higher in humans who have LDL to HDL ratios higher than those in hamsters, at least 5-fold as exemplified by normolipidemic subjects, which are not on a cholesterol-lowering medication (Table 1). While it is difficult to make any quantitative predictions, different types of studies suggest that normally, in situ biosynthesis is likely the major source of cholesterol for the human retina. For example, a study in cynomolgus monkeys showed that an atherogenic diet containing 0.8% cholesterol increased their total serum cholesterol levels from 114 mg/dl to 817 mg/dl but did not change the levels of total retinal cholesterol (51). Also, very high levels of some of the biosynthetic precursors of cholesterol were found in the retina of a spontaneously aborted fetus with a genetic defect in the last step of cholesterol biosynthesis (52). These data suggested that in situ cholesterol biosynthesis is a quantitatively important pathway in the human retina.

Perhaps the relative contributions of the pathways of retinal cholesterol input can be changed in AMD and diabetic retinopathy, diseases that affect the status of the BRBs, in which one or both become more and more compromised with disease progression (53). Yet, epidemiologic studies did not find consistent associations between the levels of serum total, LDL, and HDL cholesterol and AMD, although some recent analyses suggested that higher serum HDL levels could be associated with a higher AMD risk (54, 55). Conversely, in diabetic retinopathy, several clinical trials and epidemiological studies demonstrated a positive association between plasma LDL levels and the disease (48). In addition, recent characterization of diabetic mice (db/db) revealed that the contribution of in situ cholesterol biosynthesis to the total retinal cholesterol input was reduced to 19% during the diabetes progression as compared to 65% in control animals, while the total retinal cholesterol content was increased by 40% (56). In other words, this characterization suggested that in diabetic retinopathy, more cholesterol could enter the retina from the systemic circulation and thereby decrease the quantitative significance of in situ biosynthesis (48). Thus, the quantitative contributions of in situ biosynthesis and cholesterol uptake from the systemic circulation can vary in the retina not only between the species but even within the same species, depending on the status of the BRBs and the mechanisms and extent to which they are compromised. Perhaps other factors that still need to be established contribute as well. In any case, our findings support further investigations of cholesterol-lowering drug statins for off-label use in certain subsets of patients with AMD and diabetic retinopathy, a novel therapeutic approach, which is now considered by retinal specialists (55).

Not only disturbances in the cholesterol homeostasis in the retina but also in the brain are associated with major diseases, including Alzheimer’s and Huntington’s diseases, spinocerebellar ataxias, and amyotrophic lateral sclerosis (57, 58, 59, 60). Hence enhancing our understanding of the basic principles that govern cholesterol maintenance in the brain is important. The slower cholesterol biosynthesis and turnover rates in the brain of hamsters than in mice (Table 3) are consistent with the known interspecies differences in the brain cholesterol input (61), likely due to the difference in the ratio between the volumes of white and gray matter (62). This ratio is known to increase with an increase in brain size (62) and reflects an increase in the amount of cholesterol in the myelin pool. This pool is metabolically stable and turns over much slower than that in the metabolically active neurons, thus decreasing the overall rate of cerebral cholesterol turnover (3, 63). Our interspecies comparisons highlighted the importance of the white and gray matter volumes in determining the rate of brain cholesterol turnover, a point, which is rarely brought up and discussed in studies of brain cholesterol homeostasis.

The rate of brain cholesterol turnover also depends on sterol output, which balances cholesterol input (5, 64). Hence, we investigated cholesterol output from hamster brain and documented the correlation between the relative deuterium enrichments of the brain 24HC and brain cholesterol (Fig. 4A). We also found that ∼93% of the deuterated cholesterol pool (1/1.08, Fig. 4A equation) was converted to 24HC. Since CYP46A1, which generates 24HC, is a neuron-specific enzyme (65), this result suggests that under the experimental conditions used, brain cholesterol was probably mostly deuterated during its biosynthesis in the metabolically active cells (likely neurons), with only a small contribution from deuterium enrichment in other cell types, which eliminate cholesterol more slowly (e. g., glial cells and myelin sheaths of oligodendrocytes) (63). Further, since cholesterol 24-hydroxylation accounts for the majority of the sterol elimination from the whole brain (40%–50% in mice and 75%–85% in humans (4, 5, 62)), our data suggest that neuronal metabolism to 24HC is a significant contributor to overall cholesterol elimination from the brain.

We documented the correlation between deuterium enrichment of the brain 24HC and plasma 24HC (Fig. 4B) and established that 83% of the deuterated brain 24HC was detected in the plasma (1/1.2, Fig. 4B equation). Also, we showed that all deuterated plasma 24HC was derived from deuterated brain cholesterol (1/1, Fig. 4C equation). Thus, collectively, our studies indicated that plasma 24HC deuterium enrichment is in vivo marker for brain cholesterol biosynthesis and elimination by 24-hydroxylation and thereby brain cholesterol turnover.

Previously, plasma levels of 24HC were shown to reflect the balance between the sterol cerebral production and its hepatic metabolism to bile acids and be inversely related to the body surface (46). In addition, inflammation, dysfunction of the blood–brain barrier, state of neurodegenerative disease, and medications that alter the whole body and brain cholesterol metabolism could affect plasma 24HC levels as well (66, 67). Hence, while plasma 24HC content is a useful surrogate marker of cerebral 24HC production, caution is required while interpreting changes in the plasma 24HC levels. A more direct measure of the brain 24HC production would be helpful, and we demonstrate in the present work that deuterium enrichment of plasma 24HC could be such a measure. Moreover, we already conducted a clinical study, in which deuterated water was given to a human subject with early Alzheimer’s disease who also received low-dose efavirenz, an anti-HIV drug (68), which we found allosterically activates CYP46A1 in mouse brain (64, 69, 70). We measured plasma deuterium 24HC enrichment in this human subject during the first 8 weeks and last 6 weeks of our clinical study and found that the relative plasma 24HC deuteration was >2.5-fold higher at the end than the beginning of efavirenz administration. This result is consistent with expected efavirenz-mediated CYP46A1 activation in the brain (68) and with the data of the present work. We can now suggest that low-dose efavirenz not only activates cerebral cholesterol elimination but also cholesterol biosynthesis and turnover in the human brain. Of importance is that the plasma 24HC levels vary greatly in humans, thus making it difficult to assess the effect of the brain disease on cholesterol elimination from the brain (57). The use of the plasma relative 24HC deuteration eliminates the confounding contributions of the factors that do not relate to the CYP46A1 activity and enables a more direct comparison of cholesterol elimination and turnover in the brain between different study groups. The present work provides the necessary experimental justification for the use of deuterated water in clinical studies of brain cholesterol homeostasis.

In summary, we quantified retinal and brain pathways of cholesterol input and the brain pathway of cholesterol output by metabolism to 24HC. A comparison to mice revealed similarities and differences between the two species, thus enhancing our understanding of retinal and brain cholesterol maintenance. Studies of the brain cholesterol output were also used to establish the validity of the relative deuterium enrichment of 24HC in the plasma as in vivo marker of the CYP46A1 activity in the brain.

## Data Availability

The data that support the findings of this study are contained within the manuscript.

## Conflict of interest

The authors declare no conflict of interest.
